# Repeat sequence corresponding to the nucleosome length at the opposite end of the pairing center on autosomes in *C. elegans*

**DOI:** 10.17912/micropub.biology.001079

**Published:** 2024-02-22

**Authors:** Yukimasa Shibata

**Affiliations:** 1 Department of Biomedical Sciences, Kwansei Gakuin University

## Abstract

Many repeat sequences in genomes have unknown functions, but some have features that are suggestive of one. I found a repeat sequence that has 185 base pairs as the basic unit, which is the same as the length of a nucleosome repeat. The chromosomal location of this repeat sequence is opposite the pairing center of each autosomal chromosome. I named this repeat sequence Nucleosome Length Autosomal Repeat (NLAR). The NLAR regions reveal a low level of H3K79me, which is required for chromosome pairing. I hypothesize that NLAR inhibits chromosome pairing of autosomes from inappropriate ends during meiosis.

**Figure 1. Nucleosome Length Autosomal Repeat (NLAR):**
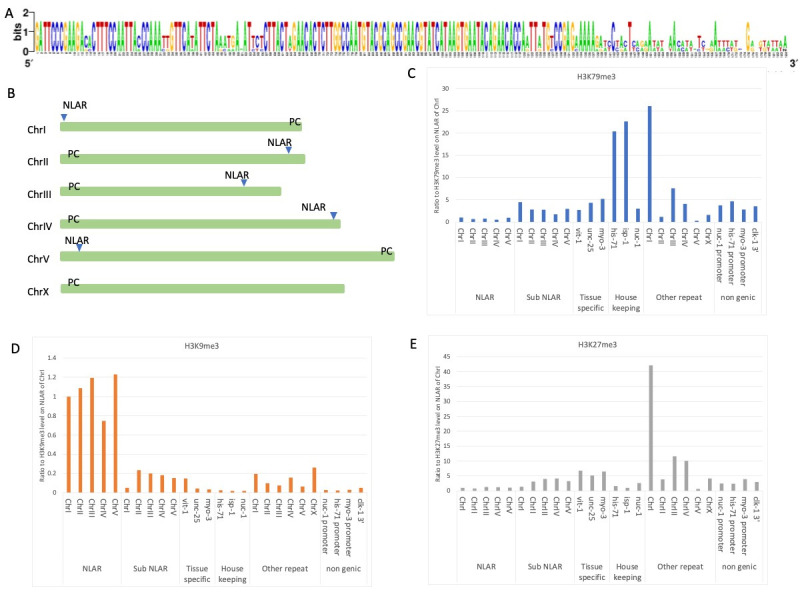
(A) Consensus sequence of NLAR. (B) Physical location of NLAR sequences; Green bar indicates each chromosome and blue arrowheads indicate the location of NLAR. PC indicates pairing center. (C-E) Graphs that represent the ratio between the average values on the indicated region and that on NLAR of ChrI. Sub NLAR regions are defined as 3kb upstream (2.5kb upstream for ChrI of H3K9) from the start and end, respectively, of each NLAR sequence. The genomic positions of the other repeats are indicated in extended data S2.

## Description

The function of particular DNA repeat sequences in the genome is largely unknown. However, the properties of the repeat's sequence, such as the length of the sequence, its position on the chromosome, and the type of epigenetic marks, sometimes suggest a function of the repeat sequence. I found a repeat sequence within autosomes of *C. elegans* with 185 base pairs (bp) as the basic unit ( Figure 1A, Extended Data S 1). Interestingly, 185 base pairs correspond to the length of the nucleosome repeat (Li and Zhu 2015) , suggesting the possibility of nucleosomes with homologous structures in the genomic regions with repeat sequences. There is one cluster of this repeat sequence on each autosome, but not on the sex chromosome ( Figure 1B ). ChrI (338441 -341228 (2787bp)), ChrII (14331913- 14323844 (8069bp)), ChrIII (11593394- 11602240 (8846bp)), ChrV (1201998- 1204599 (2601bp)) have 15, 42, 47, and 14 repeats; ChrIV (17139959-17138327, 1632 bp) has a cluster with 6 repeats of 185 bp and 4 repeats of 122 bp sequences. I named this repeat sequence Nucleosome Length Autosomal Repeat (NLAR). Each chromosome has a specific variation of the NLAR sequence. Within each chromosome, 10-20 variations were observed in the 185 bp repeat sequence that serves as the basic unit ( Figure 1A ). Comparing the standard sequence of each chromosome, there are many variations in a region within 40 bp of 185 bp, and this 40 bp region might be the linker sequence.

Each chromosome contains a pairing center, which is necessary for chromosome pairing in meiosis (Rog and Dernburg, 2013) . NLAR is located close to the opposite end of the pairing center in all autosomes ( Figure 1B ).

I searched for characteristic genomic features and epigenetic marks in the NLAR region using WormBase. The genomic region where NLAR is located has no exons and no genes are encoded in ChrI, II, III, and V. In ChrIV, NLAR is in the intron of Y116A8C.48 . The genomic region corresponding to NLAR revealed high levels of H3K9me and low levels of H3K79me and H3K27me ( Figure 1 C -E).

The highly conserved DNA sequence suggests that the chromatin structure of the NLAR region itself has a function. The inclusion of a 122 bp sequence in addition to 185 bp in ChrIV, unlike other chromosomes, may reflect that NLAR is located in an intron that undergoes transcription. I hypothesize that the chromatin structure of about 15 nucleosomes created by the NLAR inhibits the access of H3K79 methyltransferase and keeps H3K79me levels low. It is known that a mutation in *dot-1.1* that encodes H3K79 methyltransferase slows down pairing (Lascarez-Lagunas et al., 2020) . In addition, it is also known that *mrg-1* mutants show pairing defect on autosomes, although there is no pairing defect in the pairing center. Interestingly, *mrg-1* mutants do not show pairing defects on sex chromosomes (Dombecki et al., 2011), suggesting that the mechanisms that regulate chromosome pairing are different between autosomes and sex chromosomes. One possibility is that NLAR also regulates pairing of autosomes.

## Methods

To identify repeat sequences, I visually inspected the genomic sequence. Regions identified by eye to have repeating patterns were checked for the presence of repeat sequences. Since the length of NLAR corresponds to that of a nucleosome repeat, I focused on NLAR. The corresponding repeats were searched by blast through WormBase with a 185 bp sequence, which is the basic unit of NLAR. The genome sequence used was from the WS290 version of WormBase (Davis et al., 2022) . Epigenetic marks were searched using JBrowse. I performed quantitative analysis using tracks “Histone Modifications (H3K9) ChIP-chip arrays_ H3K9ME3_N2_L3” for H3K9me, “Histone Modifications (H3K79) ChIP-Seq_ AB2621_H3K79me3:361576_N2_L3” for H3K79me, and “Histone Modifications (H3K27) ChIP-Seq_ UP07449_H3K27me3:24440_N2_L3“ for H3K27me. The ratio between the average value on the indicated region and that on NLAR of ChrI was calculated. The track for H3K9 was the result of the log ratio values between amplified IP and input DNA. Therefore, the exponential of the difference between the average value on the indicated region and that on NLAR on ChrI was calculated as the ratio between the average value on the indicated region and that on NLAR of ChrI. The average score calculated from the corresponding region is indicated on the bar graph. Consensus sequences were generated using WebLogo https://weblogo.berkeley.edu/logo.cgi.

## Extended Data

Description: Complete sequence of NLAR. Resource Type: Text. DOI: 10.22002/6gnq8-4pr98

Description: List of other repeat sequence that are shown in Figure 1 C -E. Resource Type: Text. DOI: 10.22002/xawqm-87m35
